# Cognitive impairment, multimorbidity, and mobility limitations among Mexican-origin older adults

**DOI:** 10.1093/geroni/igaf122.2049

**Published:** 2025-12-31

**Authors:** Nicholas Bishop, Rebeca Wong, Fernando Riosmena, Ana Quiñones, Steven Haas

**Affiliations:** University of Arizona, Tucson, Arizona, United States; The University of Texas Health Science Center at San Antonio, San Antonio, Texas, United States; The University of Texas at San Antonio, San Antonio, Texas, United States; Oregon Health & Science University, Portland, Oregon, United States; The Pennsylvania State University, University Park, Pennsylvania, United States

## Abstract

Age-related cognitive impairment (CI), multimorbidity (MM; 2+ chronic conditions), and mobility limitations (MLs) are burdensome, develop concurrently, and lead to disablement. Examining disablement processes among Mexican-origin older adults in the US and Mexico is critical given the rapid aging and persistent health challenges faced by these groups, and migration status may modify disablement processes. Using harmonized data from the Health and Retirement Study and Mexican Health and Aging Study (2012 & 2018), we estimated cross-lagged panel models to investigate concurrent change in cognitive function (CF; average proportion of maximum score on study-specific cognitive items, 0–10), MM (count of diagnosed diabetes, hypertension, heart attack, stroke, cancer, arthritis), and MLs (count of 11 functional limitations) among Mexican-origin adults aged ≥ 65. Study/country of residence and migration status were identified (HRS: US-born [n = 215], US-immigrant [n = 173]; MHAS: non-migrant [n = 3,770], and return migrant [n = 542]). Covariates (age, sex, education, marital/partnership status, obesity, rural residence, doctor visits) were harmonized at baseline (2012). Greater baseline MM preceded increasing MLs and faster decline in CF, greater baseline MLs were associated with faster MM accumulation, and greater baseline CF was associated with slower progression of MLs. Compared to US-born respondents, MHAS respondents experienced faster accumulation of MLs and faster decline in CF, but slower MM accumulation. Greater baseline MLs were associated with faster decline in CF among the US-born, but slower decline for the other groups. These findings indicate bidirectional associations between CF, MM, and MLs, and suggest disablement processes are patterned by migration status among Mexican-origin older adults.

